# A retrospective cohort study examining secondary prevention post stroke in primary care in an Asian setting

**DOI:** 10.1186/s12875-021-01412-0

**Published:** 2021-03-25

**Authors:** Vivek Bansal, Eng Sing Lee, Helen Smith

**Affiliations:** 1 National Healthcare Group Polyclinics, 3 Fusionopolis Link, Nexus@one-north, South Tower, # 05-10, Singapore, 138543 Singapore; 2 Lee Kong Chian School of Medicine, Nanyang Technological University Singapore, Singapore, Singapore

**Keywords:** Stroke, Primary care, Neurology, Secondary prevention, Risk factors control

## Abstract

**Background:**

Stroke is one of the top contributors to burden of disability-adjusted life-years worldwide. Family physicians have key role in optimising secondary prevention following stroke by managing clinical risk factors and promoting overall control in accordance with clinical practice guidelines.

**Methods:**

Our objectives were: (i) to examine level of overall risk factor control together with control of singular risk factors one-year after an index-stroke event in individuals attending primary care facility and (ii) to describe factors associated with satisfactory risk factors control in individuals following stroke.

Study Design: Retrospective cohort study.

We conducted a study looking retrospectively at records from our electronic chronic disease database. Our study included individuals following stroke who visited primary care setting in Singapore between January 2012 to December 2016.

**Results:**

There were 24,240 individuals in our study. Overall control was better in individuals without diabetes following stroke (49.2%) as compared to those with diabetes (28.1%).

Among individuals without diabetes following stroke, factors significantly associated with overall control were sex (male) [OR (reference: female): 1.23, 95% CI: 1.10, 1.39], ethnicity (Malay) [OR (reference: Chinese): 0.72, 95% CI: 0.58, 0.90], BMI (high risk) [OR (reference: low risk): 0.72, 95% CI: 0.62, 0.84) and atrial fibrillation [OR: 1.47, 95% CI: 1.21, 1.78].

Among individuals with diabetes following stroke, factors significantly associated with overall control were sex (male) [OR (reference: female): 1.28, 95% CI: 1.12, 1.46], ethnicity (Malay) [OR (reference: Chinese): 0.81, 95% CI: 0.65, 0.99], ethnicity (Indian) [OR (reference: Chinese): 0.70, 95% CI: 0.55, 0.88], BMI (high risk) [OR (reference: low risk): 0.71, 95% CI: 0.59, 0.84), BMI (moderate risk) [OR (reference: low risk): 0.84, 95% CI: 0.72, 0.98), atrial fibrillation [OR: 1.24; 95% CI: 1.02, 1.51], chronic kidney disease [OR: 0.63, 95% CI: 0.54, 0.72] and smoking status [OR: 0.68, 95% CI: 0.54, 0.88].

**Conclusion:**

We reported sub-optimal level of overall control. Among individuals following stroke, those with diabetes had higher proportion of sub-optimal control as compared to those without diabetes. Irrespective of diabetic status, being female, having high BMI, and of Malay ethnicity as compared to Chinese ethnicity were associated with poorer overall risk factor control.

## Background

Stroke is one of the top contributors to the burden of disability-adjusted life-years (DALYs) worldwide with close to 100 million DALYs attributed to it [1]. It is the third largest contributor to the burden of DALYs within Singapore, along with other cardiovascular ailments [2, 3]. After a stroke event, individuals have an increased risk of recurrence with poorer health outcomes. The prevalence of recurrent stroke varies from 5.4% to 18.1% [4–7] and one review summarizing the prevalence of recurrent stroke across Asian settings reported first-year stroke recurrence to be up to 25.4% [8]. Recurrence is associated with adverse outcomes like increased all-cause mortality and increased risk of disability, making it important to prevent such an event from occurring [7, 9]. As more individuals are surviving the acute event, the need for optimal secondary prevention measures becomes greater. Family physicians (FP) have an important role in engaging the individuals after stroke and helping implement measures in accordance with the recommended clinical practice guidelines [10]. They have a key role in optimizing secondary prevention for individuals following stroke by managing clinical risk factors and ensuring achievement of overall control. There is evidence that prescribed lifestyle advice and medication interventions following stroke are associated with improved adherence [11].

While some studies have tried to quantify adherence to secondary prevention guidelines in the general population [12–15], not many have focused on examining the achievement of targets for secondary prevention post-stroke. Bohn et al. studied the adherence to guidelines for secondary prevention of stroke or myocardial infarction in individuals following stroke with type 2 diabetes in Germany and Austria [16]. They reported the proportion of individuals following stroke with diabetes achieving the targets for secondary prevention being 61.1%, 42.2% and 89.9% for HbA1c, LDL and blood pressure respectively. However, they did not report the proportion of individuals achieving overall control across all three parameters or risk factors, and it is this assessment of overall control which gives a more meaningful picture of secondary prevention. Another recent study from Wales reported the level of HbA1c in individuals with diabetes before and 1-year after a stroke event, with improvement in glycemic control post-stroke [17]. A US-based study reported trends in vascular risk factor control and treatment in individuals following stroke from 1999 to 2010. They reported point prevalence of control of risk factors post-stroke being 68.6%, 64.4% and 87% for blood pressure, HbA1c and cholesterol levels in men respectively [10]. Similarly, a Spanish study reported suboptimal control of risk factors in individuals following stroke, and this control was lower when compared with individuals with coronary artery disease (CAD) [18]. None of these studies were done in the Asia Pacific region, and their results are not generalizable to the Asian context due to differences in individuals’ characteristics and healthcare systems. This study was designed to address this gap.

Our study aimed to highlight the areas where secondary prevention of stroke could be improved for individuals who were on follow-up in the primary care setting following a stroke. Our specific objectives were: (i) to examine the level of overall and each singular risk factor control after index-stroke in individuals visiting primary care setting 1-year following stroke and (ii) to describe the factors associated with the achievement of overall control in these individuals 1-year following stroke.

## Methods

### Singapore healthcare system

The healthcare system in Singapore comprises of public, private, and non-profit healthcare institutions delivering a range of services including inpatient and emergency services, intermediate and long-term care (ILTC) services and outpatient services comprising of both specialist and primary care services [19]. The public sector is not only the major provider in acute care setting, covering 80% of tertiary hospitals in Singapore, but it is also central to providing primary care services to individuals with chronic ailments. Following discharge from an inpatient setting, individuals with stroke are followed-up in the community. The public primary care system in Singapore comprises of regional clusters of polyclinics which are “one-stop primary care clinics” providing a range of services including clinical, health education, diagnostic and pharmacy services [20]. The data originates from a time when there were 18 polyclinics across the island which were part of two regional clusters, and we extracted data from one of these regional clusters comprising 10 polyclinics. Given Singapore’s small size and relatively homogeneous distribution of the population, we do not expect the profile of individuals following stroke to substantially vary geographically.

Family physicians in primary care setting play a crucial role in the continuity of care and coordination of services as stroke survivors transition from an inpatient to a community setting. They bring together care professionals and providers to meet the individual’s needs and ensure that the care is integrated across various settings [21]. Continuity and coordination of care following stroke includes management of risk factors like hypertension, hyperlipidaemia and diabetes to prevent stroke recurrence and management of post-stroke mood and behaviour problems. Management of clinical risk factors (i.e., hypertension, hyperlipidaemia and diabetes) is done in accordance with the guidance provided by our Ministry of Health’s clinical practice guidelines [22], which includes initiation and titration of medications, scheduling regular follow-up appointments and monitoring laboratory test results to assess control of clinical indicators. They also give lifestyle modification advise with regards to dietary changes, smoking cessation, weight reduction and physical activity.

### Participants and setting

This was a retrospective cohort study reviewing data from the institutional electronic healthcare record database. The institutional shared electronic healthcare record facilitates provision of integrated care for patients visiting polyclinics and aids efficient monitoring of patient outcomes. This institutional electronic healthcare record database connects patients’ clinical and administrative data within a cluster of polyclinics providing summary care record including physician visits and laboratory records. Our study included individuals following stroke visiting public primary care setting in Singapore, also known as polyclinics, for follow-up with a family physician. Participants were aged 21 years and above with a past diagnosis of stroke (ICD-9 Classification of Diseases, Ninth Revision) and who received care in any of the National Healthcare Group Polyclinics (NHGP) between 1^st^ January 2012 to 31^st^ December 2016. Individuals with ischemic and haemorrhagic strokes were included as the coding of cerebrovascular accidents did not differentiate the cause, however we were able to exclude transient ischemic attacks. For participants meeting the above eligibility criteria, we retrospectively extracted information on exposure variables and outcome variables.

### Outcome or dependent variables

The outcome variables were extracted at the end of 1-year of follow-up from the baseline visit for each participant within the observation period. Our outcome variables of secondary prevention at 1-year post stroke were based on the American Heart Association (AHA) Stroke 2014 guidelines [23]. We operationalized overall control as individuals having a systolic blood pressure < 140 mmHg, and a diastolic blood pressure < 90 mmHg, LDL-cholesterol < 2.6 mmol/L and, if the individuals also had diabetes mellitus, a haemoglobin A1c (HbA1c) < 7.0%. For all individuals meeting the study eligibility criteria, we extracted the systolic and diastolic blood pressure, LDL cholesterol level and HbA1c as continuous variables. Based on the above cut-offs, these parameters were converted into binary categorical variables of ‘yes’ or ‘no’. Similarly, the recommended cut-offs for the parameters were converted to binary variables indicating whether secondary prevention targets were achieved or not. Clinical relevance and information availability in the institutional electronic healthcare record database guided the selection of outcome variables for this study and since the database did not have information on diet, physical activity and alcohol intake, these variables were not included.

### Exposure or independent variables

For all individuals meeting the study eligibility criteria, we extracted socio-demographic and clinical variables at the baseline visit to the polyclinic. Sociodemographic data included age (continuous variable in years), sex (categorical variable with two categories of ‘male’ and ‘female’), ethnicity (four categories of ‘Chinese’, ‘Malay’, ‘Indian’ and ‘Others’). Clinical data included body mass index (BMI) (continuous variable converted to categorical variable based on the Asian cut-off with three categories of ‘low’ (less than 23 kg/m^2^), ‘moderate’ (23 to 27.4 kg/m^2^) and ‘high’ (more than 27.4 kg/m^2^) risk) and smoking status (categorical variable with two categories of ‘yes’ or ‘no’). In addition, we also captured the presence of the following comorbid clinical conditions (as binary categorical variables of ‘yes’ or ‘no’): hyperlipidemia, hypertension, diabetes, chronic kidney disease (CKD), ischemic heart disease (IHD), and atrial fibrillation (AF). Clinical relevance and information availability in the institutional electronic healthcare record database guided the selection of above mentioned cardiometabolic comorbid conditions.

### Analysis

We used de-identified data to conduct our analysis. We used descriptive analysis to summarize our categorical variables using proportions and frequencies. Complete case analysis was used to study the implementation of treatment goals for secondary prevention in individuals with a prior history of stroke. To describe the factors associated with the achievement of overall control in individuals following stroke, we performed multivariable logistic regression analysis since our outcome variable was binary (overall control achieved versus not), and we controlled for the following pre-decided medically relevant variables: age, sex, ethnicity, BMI, smoking status, presence of IHD, presence of AF and presence of CKD. All the analysis was performed using STATA version14.2, and the statistical significance level was set at 5%.

#### Sample size

As per our original intention, we selected all available individuals from Institutional electronic healthcare record database between the period of time from 1^st^ January 2012 to 31^st^ December 2016, with a past diagnosis of stroke (ICD-9 Classification of Diseases, Ninth Revision) and who received care in any of the National Healthcare Group Polyclinics (NHGP). The sample extracted was 24,240 individuals. Choosing the level of confidence of 95%, 0.05 precision level, expected prevalence or proportion of individuals following stroke meeting overall control target (as per clinical practice guidelines) being set at 50%, our sample size meets the minimum requirement to observe a significant effect [24, 25].

## Results

We have summarized the basic socio-demographic information at baseline for all the included participants in Table 1. With regards to age, 58.9% of the participants were 65 years old and above while 38.7% were between age of 45 to 64 years old. With regards to sex, 57.7% of the participants were male and 42.3% were female. With regards to race, majority were Chinese (80.1%), followed by Malay (10.8%), Indian (6.5%) and others (2.6%). About 41.4% had a body mass index (BMI) between 23.0 to 27.4 kg/m^2^ (‘moderate’ risk). Hyperlipidemia was the most common chronic condition (98.1%) followed by hypertension (92.9%), diabetes (54.9%), CKD (chronic kidney disease) (54.1%), IHD (Ischemic heart disease) (20.1%) and AF (Atrial fibrillation) (12.3%).

**Table 1:** Baseline characteristics of participants (*N* = 24,240)

**Variable**	**N**^**a**^** (%)**
Age (in years)	
Less than 45	572 (2.4%)
45 to 64	9,393 (38.7%)
65 and above	14,275 (58.9%)
Sex	
Female	10,258 (42.3%)
Male	13,982 (57.7%)
Race	
Chinese	19,438 (80.1%)
Malay	2,613 (10.8%)
Indian	1,570 (6.5%)
Others	619 (2.6%)
Body Mass Index (kg/m^2^)	
Less than 23	4,476 (31.0%)
23 to 27.4	5,968 (41.4%)
More than 27.4	3,983 (27.6%)
Smoking status	
Current smoker	1,624 (6.7%)
Comorbid conditions(yes)	
Hyperlipidemia	23,779 (98.1%)
Hypertension	22,525 (92.9%)
Diabetes	13,307 (54.9%)
Chronic kidney disease^b^	13,104 (54.1%)
Ischemic heart disease	4,877 (20.1%)
Atrial fibrillation	2,990 (12.3%)

Data from individuals seen at polyclinics from 1^st^ Jan 2012 to 31st Dec 2016 with a diagnosis of stroke coded. Total sample size (*N* = 24,240)

^a^ All numbers may not add up to total because of missing data

^b^ those individuals with documented eGFR (estimated glomerular filtration rate) value less than 60 ml/min/1.73m2

Table 2 shows the proportion of individuals at 1-year following stroke with optimally controlled hypertension, diabetes, and hyperlipidemia as 71.4%, 52.9%, and 66.6% respectively. Among individuals who had complete data, overall control was better in individuals without diabetes following stroke (49.2%) as compared to individuals with diabetes following stroke (28.1%).

**Table 2:** Risk factor control profile at 1-year following stroke

**Risk Factor (each singular or composite)**	**Numerator**	**Proportion (%)**
Overall Control (Non-Diabetic)^a^	3,195	49.2%
Overall Control (Diabetic)^b^	1,964	28.1%
Blood Pressure Control^c^	13,934	71.4%
Lipids (LDL) Control^d^	9,804	66.6%
Glycaemic Control^e^	4,782	52.9%

Based on the American Heart Association/ American Stroke Association (AHA/ASA) Stroke 2014 guidelines [18], we operationalized our outcome variable of secondary prevention post-stroke as overall control which comprised of individuals having a systolic blood pressure < 140 mmHg and a diastolic blood pressure < 90 mmHg, LDL-cholesterol < 2.6 mmol/L, and haemoglobin A1c (HbA1c) < 7% (for diabetes mellitus) for those individuals with diabetes following stroke

^a^ For the individuals without diabetes following stroke, only 6,501 had readings available for both blood pressure and LDL

^b^ For the individuals with diabetes following stroke, only 6,999 individuals had readings available for all three (HbA1c, blood pressure and LDL)

^c^ Out of the 22,525 individuals with hypertension, only 19,529 had blood pressure readings available

^d^ Out of the 23,779 individuals with hyperlipidaemia, only 14,714 had LDL readings available

^e^ Out of the 13,307 individuals with diabetes, only 9,046 had HbA1c readings available. Glycaemic control is determined by the level of HbA1c

Table 3 shows the multivariable regression analysis results for individuals without diabetes following stroke, demonstrating the association of socio-demographic and clinical variables with overall control. Among those individuals without diabetes following stroke, sex, ethnicity, BMI and AF were significantly associated with overall control. With regards to sex, being male increased the odds of overall control by 1.23 (CI 1.10, 1.38) times as compared to females With regards to ethnicity, Malay had 0.72 (CI: 0.58, 0.90) times the odds of achieving overall control as compared to Chinese ethnicity. Compared to the low-risk category of BMI, those in the high-risk category had 0.72 (CI: 0.62, 0.84) times the odds of overall control. Having AF increased the odds of overall control by 1.46 (CI: 1.21, 1.78) times when compared to those without AF. To summarize, among those individuals without diabetes following stroke, factors associated with poor overall control were Malay ethnicity and having higher BMI. Factors associated with better overall control were being male and presence of AF.

**Table 3:** Results for individuals without diabetes following stroke, association of socio-demographic and clinical variables with overall control

Overall Control	OR (95% CI)	*P*-value	aOR (95% CI)	*P*-value
**Age (in years)**				
Less than 45	Ref		Ref	
45 to 64	0.80 (0.59, 1.09)	0.162	0.88 (0.63, 1.24)	0.759
65 and above	0.87 (0.64, 1.18)		0.88 (0.62, 1.25)	
**Sex**				
Female	Ref		Ref	
Male	1.20 (1.09, 1.32)	< 0.001	1.23 (1.10, 1.38)	**0.001**
**Race**				
Chinese	Ref		Ref	
Malay	0.74 (0.61, 0.89)		0.72 (0.58, 0.90)	
Indian	0.89 (0.68, 1.15)	0.012	0.93 (0.69, 1.25)	**0.040**
Others	0.93 (0.67, 1.29)		0.95 (0.66, 1.38)	
**Body Mass Index** (kg/m^2^)				
Less than 23	Ref		Ref	
23 to 27.4	0.92 (0.81, 1.04)	< 0.001	0.92 (0.81, 1.04)	** < 0.001**
More than 27.4	0.70 (0.60, 0.81)		0.72 (0.62, 0.84)	
**Smoking status**				
No	Ref		Ref	
Yes	0.91 (0.76, 1.09)	0.287	0.91 (0.75, 1.11)	0.365
**Ischemic heart disease**				
No	Ref		Ref	
Yes	1.04 (0.90, 1.19)	0.621	0.92 (0.78, 1.08)	0.290
**Atrial Fibrillation**				
No	Ref		Ref	
Yes	1.53 (1.30, 1.81)	< 0.001	1.46 (1.21, 1.78)	** < 0.001**
**Chronic Kidney Disease**				
No	Ref		Ref	
Yes	0.96 (0.87, 1.06)	0.451	0.91 (0.81, 1.03)	0.131

*Abbreviations*: *OR* Odds ratio, *aOR* Adjusted odds ratio, *CI* Confidence interval, *Ref* Reference value

Table 4 shows the multivariable regression analysis results for individuals with diabetes following stroke, demonstrating the association of socio-demographic and clinical variables with overall control. Among those individuals with diabetes following stroke, sex, ethnicity, BMI, AF, chronic kidney disease and smoking status were significant covariates. With regards to sex, being male increased the odds of overall control by 1.28 (CI: 1.12, 1.46) times as compared to females. With regards to ethnicity, Malay and Indian had 0.81 (Cl: 0.65, 0.99) and 0.70 (Cl: 0.55, 0.88) times the odds of having overall control as compared to Chinese ethnicity respectively. Compared to the low-risk category of BMI, those in the moderate and high-risk category had 0.84 (Cl: 0.72, 0.98) and 0.71 (Cl: 0.59, 0.84) times the odds of overall control. Having AF increased the odds of overall control by 1.24 (Cl: 1.02, 1.52) times when compared to those without AF. Having CKD decreased the odds of overall control by 0.63 (Cl: 0.54, 0.72) times when compared to those without CKD. Being a smoker decreased the odds of control by 0.68 (Cl: 0.54, 0.88) times as compared to a non-smoker. To summarize, among those individuals with diabetes following stroke, factors associated with poor overall control were Malay or Indian ethnicity, having higher BMI, CKD and being a smoker. Factors associated with better overall control were being male and presence of AF.

**Table 4:** Results for individuals with diabetes following stroke, association of socio-demographic and clinical variables with overall control

Overall Control	OR (95% CI)	*P*-value	aOR (95% CI)	*P*-value
**Age (in years)**				
Less than 45	Ref		Ref	
45 to 64	0.84 (0.55, 1.28)	0.001	0.75 (0.46, 1.22)	0.068
65 and above	1.02 (0.67, 1.56)		0.87 (0.53, 1.42)	
**Sex**				
Female	Ref		Ref	
Male	1.24 (1.11, 1.37)	< 0.001	1.28 (1.12, 1.46)	** < 0.001**
**Race**				
Chinese	Ref		Ref	
Malay	0.70 (0.59, 0.83)		0.81 (0.65, 0.99)	
Indian	0.67 (0.54, 0.82)	< 0.001	0.70 (0.55, 0.88)	**0.009**
Others	0.81 (0.56, 1.17)		0.87 (0.56, 1.35)	
**Body Mass Index** (Kg/m^2^)				
Less than 23	Ref		Ref	
23 to 27.4	0.83 (0.71, 0.96)	< 0.001	0.84 (0.72, 0.98)	** < 0.001**
More than 27.4	0.65 (0.55, 0.77)		0.71 (0.59, 0.84)	
**Smoking status**				
No	Ref		Ref	
Yes	0.73 (0.59, 0.91)	0.005	0.68 (0.54, 0.88)	**0.003**
**Ischemic heart disease**				
No	Ref		Ref	
Yes	0.93 (0.83, 1.06)	0.282	0.90 (0.77, 1.05)	0.177
**Atrial Fibrillation**				
No	Ref		Ref	
Yes	1.20 (1.02, 1.41)	0.024	1.24 (1.02, 1.52)	**0.028**
**Chronic Kidney Disease**				
No	Ref		Ref	
Yes	0.69 (0.61, 0.77)	< 0.001	0.63 (0.54, 0.72)	** < 0.001**

*Abbreviations*: *OR* Odds ratio, *aOR* Adjusted odds ratio, *CI* Confidence interval, *Ref* Reference value

## Discussion

In this study, we estimated the proportion of individuals following stroke whose secondary prevention was optimal over 1-year following stroke and described the characteristics of these individuals following stroke. Optimal secondary prevention was recorded in 28.1% of individuals with diabetes following stroke and 49.2% of those without diabetes. In individuals with diabetes following stroke, we found that sex, ethnicity, BMI, smoking, AF, and CKD were significantly associated with the achievement of overall control. While in individuals without diabetes following stroke, we found sex, ethnicity, BMI and AF were significantly associated with the achievement of overall control. Irrespective of the diabetes status, being female, having high BMI, and of Malay ethnicity were associated with poor overall control.

### Comparisons with other studies

Published studies tend to focus on singular risk factor reduction rather than the overall control achieved. Comparing our results with single risk factor studies, we found examples of some study populations doing better and others worse. For example, comparing the proportion of individuals following stroke having an optimal level of blood pressure, two studies reported a higher level of control at 89.9% [16], and 86% [26], as compared to our estimate of 71.4%. The second of these studies was a prospective study from Canada in which stroke prevention clinics at a tertiary care setting recruited 119 individuals who were referred to them from primary care for secondary stroke prevention. The relatively higher proportion of individuals with blood pressure control could be due to their study setting being a specialized stroke prevention clinic (as compared to primary care setting in our study) and only a small proportion of all participants in this study had a recent history of stroke (as compared to all participants in our study) [26]. The possible explanation for a higher proportion of participants achieving blood pressure control in the other study may be due to the longer 10-year follow-up period compared to our 1-year follow-up. Moreover, all the participants with stroke in this study had a history of diabetes [16]. Those studies reporting a lower level of blood pressure control as compared to our estimate reported values ranging from 23.8% to 62.4% [10, 18, 27–29]. Possible explanation for differences could be related to different healthcare systems, care settings and patient characteristics.

Comparing the level of lipid control across different studies, all the reviewed studies reported a lower level of lipid control as compared to our estimate of 66.6%, with reported estimates ranging from 13.9% to 49.0% [16, 18, 26, 28, 29]. We found 52.9% of individuals with diabetes following stroke achieving the target level of glycemic control. Compared to existing literature, two studies reported a higher proportion of glycaemic control [10, 16], and another two reported a lower proportion of glycaemic control [18, 26]. The possible explanation for this difference could be related to the cut-off value used for HbA1c which was 7% in our study (as compared to 7.5% in this compared study) [16]. As for the other study, it included participants from a national survey who self-reported history of stroke [10] and these may be systematically different from the participants in our retrospective record-based study.

Our study filled an important gap in current literature as a limited number of current studies address the level of secondary prevention attained. Among the studies reviewed, only two attempted to report a composite or combined estimate of control of risk factors achieved, with one reporting 3.3% of individuals with ischemic stroke achieving control of all risk factors [18]. Another study reported the proportion of individuals following stroke achieving control of both blood pressure and lipids to be about 19.4% [29]. While our study reported more encouraging levels of secondary prevention achieved, as compared to the above two studies, there remains considerable room for improvement. The reasons for not achieving the optimal level of control of risk factors could be multiple. These could be at the patient level, the physician level, the health system level, or a combination of these. A study reported that in spite of 90% of individuals following stroke being on specific drug regimens, only about one-fourth of them achieved the recommended risk factor control [18]. This highlights the complexity of addressing secondary control following stroke. For example, the individual’s adherence to medication, compliance to lifestyle factors or healthcare system factors such as difficulty in accessing services or financial barriers, can all play a part. Another study based in Canada reported poor control of risk factors after either a coronary artery disease (CAD) or cerebrovascular disease (CVD) [29]. Those with CVD had worse control of risk factors as compared to those with CAD, with the former group having 46.0%, 40.5% and 19.4% of post-individuals with CVD meeting target levels of blood pressure, LDL and both respectively. In spite of good adherence to secondary prevention guidelines (medication rates ranging from 76.5% to 91.3%), it did not translate to the achievement of risk factor control suggesting the importance of other elements like patient factors.

We reported lower overall control in individuals with diabetes following stroke as compared to individuals without diabetes following stroke. There are literature that support poorer control of other risk factors in individuals following stroke who have diabetes. Individuals with diabetes were associated with lower odds (OR = 0.16; 95% CI: 0.14, 0.19) of achieving the target blood pressure level compared to individuals with a previous cerebrovascular event [29]. Another study reported lower levels of control of lipids in individuals with diabetes [16]. Another possible explanation could be the difficulty managing co-occurrence of multiple chronic conditions experienced by both healthcare providers and patients.

Our finding was in agreement with other studies that showed, a significant association between sex and achievement of target levels of risk factors [16, 29]. One such study reported the largest difference across males and females in the achievement of the target level of serum LDL levels, with 46.1% of men and 38.3% of women achieving the target [16]. It is important to further study these sub-groups of individuals following stroke to intervene in an evidence-based manner and promote optimal secondary prevention since diabetes itself is an independent predictor of recurrent stroke with about 9.1% of stroke cases being attributable to it [30–32].

Focusing on the modifiable factors found to be significantly associated with overall control post-stroke, following are some implications for practice arising from our current work. We recommend that efforts and resources should be directed towards developing adequate weight management services and smoking cessation services within primary care setting. The family physicians should adopt a holistic approach to secondary prevention post-stroke with incorporation of both pharmacological and non-pharmacological management in their care provision. There should be relatively more focus on lifestyle modification advice and guidance with regards to dietary changes, weight reduction and physical activity. At the health system level, provision of financial subsidies aimed at weight management and smoking cessation services will increase the delivery and uptake of these services. At provider level, directing adequate resources to develop capacity for delivering such services within primary care setting would be beneficial.

### Strengths and limitations of this study

Our study has several strengths including the large sample size from 10 polyclinics over a period of 5 years. We captured major risk factors associated with recurrent stroke with a large database. Compared to observational study design which includes self-reported data, our study has the advantage of physician recorded data from electronic health records. This was one of the few studies to provide estimates of the overall control of risk factors post-stroke in an Asian setting, and we have added new knowledge to the existing literature on the prevalence of control of each singular risk factor.

The study also has several limitations. The database could not provide the causation of stroke (ischemic versus haemorrhagic) experienced by each individual, which may influence treatment recommendations by clinicians. Moreover, the database did not have information on other relevant variables such as the functional status of the individuals following stroke, education level, employment status, diet, physical activity, alcohol intake and available psychosocial support. Another limitation was related to missing data, for which we opted to conduct complete case analysis. Another shortcoming was that we could only assess the proportion of individuals meeting or not meeting the treatment goals but could not elicit the reasons why. Qualitative research exploring the experiences of individuals following stroke and their caregivers engaging in secondary prevention related behaviours will be needed. Our focus was on the overall and singular risk factor control at 1-year for individuals following stroke in the primary care setting. We did not explore the relative difference between baseline and 1 year following a stroke.

## Conclusion

Optimizing secondary prevention post-stroke should be made a priority given the burden of stroke to our healthcare system. Among individuals following stroke, those with diabetes had a higher proportion of sub-optimal control as compared to those without diabetes. Irrespective of the diabetes status, being female, having high BMI, and of Malay ethnicity were associated with poor overall control and interventions targeting these sub-groups might be helpful. Highlighted gaps represent a significant missed opportunity for the prevention of adverse events in individuals at high risk for a recurrent stroke. Future research efforts should focus on exploring the association of overall control with outcomes such as stroke recurrence or other cardiovascular events.

## Data Availability

The datasets used and/or analysed during the current study are available from the corresponding author on reasonable request.
